# Available Bleeding Scoring Systems Poorly Predict Major Bleeding in the Acute Phase of Pulmonary Embolism

**DOI:** 10.3390/jcm10163615

**Published:** 2021-08-16

**Authors:** Camille Mathonier, Nicolas Meneveau, Matthieu Besutti, Fiona Ecarnot, Nicolas Falvo, Benoit Guillon, François Schiele, Romain Chopard

**Affiliations:** 1 Department of Cardiology, University Hospital Jean Minjoz, 25000 Besançon, France; camille.mathonier@gmail.com (C.M.); nicolas.meneveau@univ-fcomte.fr (N.M.); mbesutti.univ@gmail.com (M.B.); fiona.ecarnot@univ-fcomte.fr (F.E.); benoit.guillon@univ-fcomte.fr (B.G.); francois.schiele@univ-fcomte.fr (F.S.); 2 EA3920, University of Burgundy Franche-Comté, 25000 Besançon, France; 3 F-CRIN, INNOVTE Network, CHU de Saint-Étienne-CIC 1408, Hôpital Nord-Médecine Vasculaire et Thérapeutique, CEDEX 2, 42055 Saint-Etienne, France; 4 Department of Vascular Medicine, University Hospital of Dijon, 21079 Dijon, France; nicolas.falvo@chu-dijon.fr

**Keywords:** pulmonary embolism, bleeding, scores

## Abstract

We aimed to compare six available bleeding scores, in a real-life cohort, for prediction of major bleeding in the early phase of pulmonary embolism (PE). We recorded in-hospital characteristics of 2754 PE patients in a prospective observational multicenter cohort contributing 18,028 person-days follow-up. The VTE-BLEED (Venous Thrombo-Embolism Bleed), RIETE (Registro informatizado de la enfermedad tromboembólica en España; Computerized Registry of Patients with Venous Thromboembolism), ORBIT (Outcomes Registry for Better Informed Treatment), HEMORR_2_HAGES (Hepatic or Renal Disease, Ethanol Abuse, Malignancy, Older Age, Reduced Platelet Count or Function, Re-Bleeding, Hypertension, Anemia, Genetic Factors, Excessive Fall Risk and Stroke), ATRIA (Anticoagulation and Risk Factors in Atrial Fibrillation), and HAS-BLED (Hypertension, Abnormal Renal/Liver Function, Stroke, Bleeding History or Predisposition, Labile International Normalized Ratio, Elderly, Drugs/Alcohol) scores were assessed at baseline. International Society on Thrombosis and Haemostasis (ISTH)-defined bleeding events were independently adjudicated. Accuracy of the overall original 3-level and newly defined optimal 2-level outcome of the scores were evaluated and compared. We observed 82 first early major bleedings (3.0% (95% CI, 2.4–3.7)). The predictive power of bleeding scores was poor (Harrel’s C-index from 0.57 to 0.69). The RIETE score had numerically higher model fit and discrimination capacity but without reaching statistical significance versus the ORBIT, HEMORR_2_HAGES, and ATRIA scores. The VTE-BLEED and HAS-BLED scores had significantly lower C-index, integrated discrimination improvement, and net reclassification improvement compared to the others. The rate of observed early major bleeding in score-defined low-risk patients was high, between 15% and 34%. Current available scoring systems have insufficient accuracy to predict early major bleeding in patients with acute PE. The development of acute-PE-specific risk scores is needed to optimally target bleeding prevention strategies.

## 1. Introduction

Anticoagulation is the cornerstone of the treatment of pulmonary embolism (PE) and should be initiated promptly when PE is diagnosed or a high clinical suspicion exists. Anticoagulant therapy aims to reduce mortality, morbidity of thrombus extension, and recurrence [1]. Moreover, 3% of patients present with a high-risk PE, and around 5% of intermediate-risk PE patients who develop secondary hemodynamic collapse require emergent advanced therapies, mostly by infusing systemic thrombolysis [2,3,4]. 

Bleeding events are the main drawback of antithrombotic therapies. Cohort studies report that the mortality linked to major bleeding events is up to 20%, i.e., twice as high as the rate of death from recurrent PE [5]. Major bleeding was identified as a predictor of short and 1-year mortality [5,6,7] and occurred more frequently within the first 7 days [5,8]. 

A cohort study evaluating the impact of long-term dose adjustment of direct oral anticoagulants (DOACs) previously reported that physicians in charge were intuitively aware of patients’ bleeding risk in the acute phase of PE [9]. However, more standardized and reproducible approaches using bleeding scoring systems have been developed, which may help to define the optimal antithrombotic management. Two bleeding risk-prediction scores for patients with venous thromboembolism (VTE), RIETE (Registro informatizado de la enfermedad tromboembólica en España; Computerized Registry of Patients with Venous Thromboembolism) and VTE-BLEED (Venous Thrombo-Embolism Bleed) scores, have been proposed and externally validated [10,11]. In addition, several bleeding scores for patients with atrial fibrillation (AF) are available (e.g., ORBIT (Outcomes Registry for Better Informed Treatment), HEMORR_2_HAGES (Hepatic or Renal Disease, Ethanol Abuse, Malignancy, Older Age, Reduced Platelet Count or Function, Re-Bleeding, Hypertension, Anemia, Genetic Factors, Excessive Fall Risk and Stroke), ATRIA (Anticoagulation and Risk Factors in Atrial Fibrillation), and HAS-BLED (Hypertension, Abnormal Renal/Liver Function, Stroke, Bleeding History or Predisposition, Labile International Normalized Ratio, Elderly, Drugs/Alcohol) scores [12,13,14,15]. All these scores were built to assess bleeding risk in stable patients receiving long-term anticoagulation. Currently, few data are available regarding their ability to predict in-hospital major bleeding in the context of acute PE [6,16]. 

Therefore, we aimed to externally validate and compare the predictive value of the RIETE, VTE-BLEED, ORBIT, HAEMORR_2_HAGES, ATRIA, and HAS-BLED bleeding scores for the occurrence of major bleeding during the hospital stay of acute PE patients. 

## 2. Materials and Methods

This cohort study is a non-interventional retrospective post hoc analysis based on prospectively collected data from five French centers (two tertiary care facilities and three general hospitals) between January 2011 and September 2019 and recorded in the BFC-FRANCE registry [17]. This registry received approval from the national commission for data privacy and protection. This study was conducted in accordance with the amended Declaration of Helsinki. All patients provided written informed consent, and our institutional review board approved the study. We report the study methods and results in accordance with the STrengthening the Reporting of OBservational studies in Epidemiology (STROBE) guidelines [18]. 

### 2.1. Patients and Setting

We prospectively recorded all consecutive patients ≥18 years with a confirmed diagnosis of PE by computed tomography pulmonary angiography (CTPA) or ventilation-perfusion (V–Q) scan. For confirmation of the diagnosis of PE, we required an intraluminal filling defect on CTPA [19], or a high probability V–Q scan according to the prospective investigation of pulmonary embolism diagnosis (PIOPED) criteria [20]. There were no exclusion criteria. Management was at the discretion of the physician in charge and was in accordance with current guidelines [3,21,22]. Anticoagulant therapy included parenteral anticoagulant (i.e., unfractionated heparin, low molecular weight heparin, and fondaparinux) and oral anticoagulant (i.e., vitamin K antagonist (VKA) and direct oral anticoagulant (DOAC)). Reperfusion therapy included systemic thrombolysis and surgical embolectomy. Pulmonary embolism was risk stratified according to the European Society of Cardiology (ESC) guidelines [3].

### 2.2. Bleeding Definition

Early bleeding was defined as a bleeding event that occurred during the hospital stay (i.e., between PE diagnosis and hospital discharge). Major bleeding was defined according to the definition proposed by the Control of Anticoagulation Subcommittee of the International Society on Thrombosis and Hemostasis (ISTH): (1) fatal bleeding, and/or (2) symptomatic bleeding in a critical area or organ, such as intracranial, intraspinal, intraocular, retroperitoneal, intra-articular, pericardial, or intramuscular with compartment syndrome, and/or (3) bleeding causing a drop of hemoglobin level of 20 g/L or more, or leading to transfusion of two or more units of red blood cells [23]. All bleeding events were classified by a central adjudication committee (CM and RC). An independent data safety monitoring board periodically reviewed the outcome. Disagreement was resolved by a third author (NM).

### 2.3. Bleeding Predicting Scores

Based on a critical review of the literature, six different bleeding prediction scores were selected and calculated in all study patients at baseline: the RIETE score [11], the VTE-BLED score [10], the ORBIT score [15], the HAEMORR_2_HAGES score [14], the ATRIA score [12], and the HAS-BLEED score [13]. All scores but one, were calculated prospectively (the VTE-BLED score was developed in 2016 and was calculated retrospectively between 2011 and 2016). Since CYP 2C9 single-nucleotide polymorphisms were not assessed as part of this study, all patients were scored 0 points for this item in the HEMORR_2_HAGES [14] score. For calculation of the HAS-BLED score, all patients were scored with 0 points for ‘‘labile INR’’ since therapeutic anticoagulation with VKA was not initiated yet at baseline [13]. ORBIT, HEMORR_2_HAGES, ATRIA, HAS-BLED, VTE-BLEED, and RIETE scores and staging systems for risk of major bleeding complications are provided in Supplementary Table S1.

### 2.4. Statistical Analysis

Continuous variables are expressed as mean ± standard deviation. Categorical variables are expressed as number (percentage). Unadjusted differences between patients who experienced in-hospital major bleeding and those who did not were compared using the chi-square test for categorical variables and Student’s *t*-test for continuous variables. The use of multiple imputation was not required as the rate of missing data was <1% for all covariates [24]. The potential for covariate multiple collinearity was tested using the variance inflation factor (VIF) and condition number (CN), with VIF < 10 and CN < 30 as reference values [25]. The cumulative rate of a first major bleeding event was illustrated using the Kaplan–Meier method. Independent predictors of in-hospital major bleeding, in-hospital mortality, and length of stay were determined by multivariable logistic regression, adjusted for baseline characteristics, and in-hospital therapies that yielded a *p* value < 0.10 by univariable analysis. Results are reported as odds ratio (OR) with 95% confidence interval (CI). The relationship between dichotomized bleeding risk scores and in-hospital major bleeding was also analyzed with logistic regressions. 

The global model fit of the six bleeding risk scores was assessed by calculation of Nagelkerke’s R^2^, the Bayes information criterion (BIC), and the Akaike information criterion (AIC). Discrimination of models was evaluated by Harrell’s C-index [26]. Receiver operating characteristics (ROC) curves illustrated discriminative capacities of each model. Sensitivity, specificity, positive predictive value (PPV), and negative predictive value (NPV) of each model were derived from the ROC curves. Model calibrations were assessed visually by plotting the mean of model-predicted in-hospital major bleeding in each decile of predicted in-hospital major bleeding against the observed in-hospital major bleeding estimated by the Kaplan–Meier method. The original RIETE, ORBIT, HAEMORR_2_HAGES, ATRIA, and HAS-BLEED scores used 3-level categories: low-, intermediate- and high-risk. The RIETE score did not classify any patient as low-risk since all patients received one point for PE diagnosis. The VTE-BLED score was not developed as a 3-level category. To enable a more practical 2-level interpretation, i.e., low- and high-risk categories, an optimal threshold was determined by ROC curve analyses. 

To determine the most accurate bleeding risk model for the prediction of in-hospital major bleeding in acute PE, we compared Harrell’s C-indices using the approach proposed by Kang et al. as well as the net reclassification improvement (NRI) and the integrated discrimination improvement (IDI) between models [27,28]. To assess the robustness of the findings, we performed sensitivity analyses by estimating the c-indices of the bleeding scores in the following subgroups: (1) non-high risk PE patients, and (2) patients who did not receive reperfusion therapy (i.e., systemic thrombolysis or surgical embolectomy).

A *p* value < 0.05 was considered significant. Analyses were performed using SAS 9.4 (SAS institute Inc., Cary, NC, USA).

## 3. Results

In total, 2757 patients were admitted to the participating centers during the study period with an objective diagnosis of acute PE. In-hospital data were not recorded for 3 patients (0.1%). The remaining 2754 patients comprised the study population. Mean age was 67.3 ± 17.4 years; 1414 (51.3%) were women. One hundred and thirty-three patients (4.8%) had high-risk PE, 584 patients (21.2%) intermediate-high risk PE, 1594 patients (57.9%) intermediate-low risk PE, and 443 patients (16.1%) low-risk PE.

### 3.1. Major Bleeding Events

**Figure 1:**
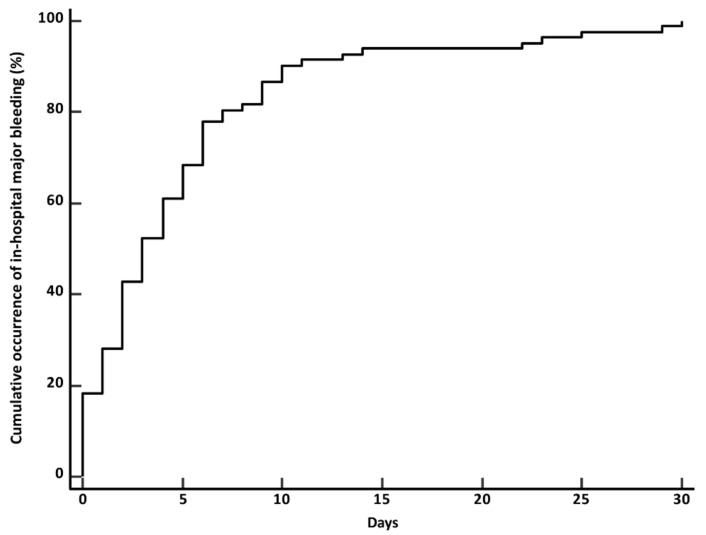
Cumulative rate of a first major bleeding event.

During the in-hospital stay, 82 patients (3.0%; 95% CI, 2.4–3.7) had major bleeding with a median time to event of 2.8 days (Q1–Q3, 1.2–3.9, ranging from 1 to 21 days). Figure 1 illustrates the cumulative time from admission to major bleeding. Bleeding events were classified according to the ISTH definition as major because of the occurrence of at least one of the following criteria: bleeding-related death, 9 patients (10.9%), symptomatic bleeding in a critical area or organ, 28 patients (34.1%), bleeding requiring surgery, 13 patients (15.8%), bleeding causing a fall in hemoglobin level of 2.0 g/dL, 58 patients (70.7%), and bleeding leading to transfusion of two or more units of whole blood or red cells, 48 patients (58.5%). Bleeding in a critical area or organ was intracranial for 18 patients (0.6%), intraspinal for 1 patient (0.03%), intraocular for 1 patient (0.03%), retroperitoneal for 2 patients (0.06%), and intramuscular for 6 patients (0.21%).

Overall, patients who suffered early bleeding were more frequently women, had a more severe hemodynamic profile, RV dysfunction, and positive troponin resulting in a higher sPESI (simplified Pulmonary Embolism Severity Index) and more severe ESC-defined risk stratification (Table 1). Among patients with a bleeding event, 1.2% were treated with DOAC, 19.5% with VKA, 68.3% with parenteral anticoagulant, and 11.0% with systemic thrombolysis. Concomitant medication usage predisposing to bleeding, syncope, heart rate > 80 b.p.m, renal dysfunction and anemia at admission were factors related to early major bleeding after multivariable adjustment (Table 2). The occurrence of early major bleeding was related to a longer adjusted length of stay (OR, 4.2; 95% CI, 2.9–5.8) and a higher rate of adjusted in-hospital mortality (OR, 8.4; 95% CI, 4.0–17.6) (Table 2).

### 3.2. Bleeding Scores and Prediction of Early Major Bleeding

Median (Q1–Q3) bleeding risk scores in the study population were as follows: RIETE, 3 (2–4); VTE-BLEED, 2.5 (1.5–3.5); ORBIT, 1 (0–2); HAEMORR_2_HAGES, 1 (0–1); ATRIA, 1 (0–3); and HAS-BLED, 1 (0–1) (Table 1). Using the original 3-level risk categories, the ORBIT, HAEMORR_2_HAGES, and ATRIA scores classified the majority of patients in the low-risk category (70.5–86.1%) whereas the RIETE and the HAS-BLED classified the majority of patients in the intermediate category (78.1% and 65.5%, respectively). All but one (i.e., the ORBIT score) were able to categorize patients with increasing rates of observed early major bleeding across the 3-level categories. The rate of observed major bleeding ranged between 1.8% and 2.4% in the low-risk categories, 2.3% and 7.7% in the intermediate-risk categories, and 5.3% and 7.0% in the high-risk categories. After dichotomization of the six bleeding scores into 2-level risk categories, all scores were able to distinguish between low and high risk of observed early major bleeding (Table 3). 

**Figure 2:**
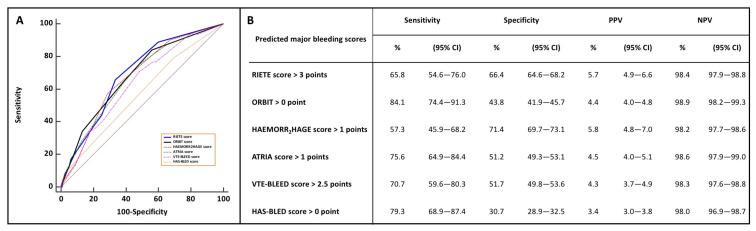
Receiving operator curves analyses of the six-bleeding risk scores (**A**) and their corresponding sensitivity, specificity, positive predicting value, and negative predictive value based on the adjusted-threshold 2-level categories (**B**). PPV: positive predictive value; NPV: negative predictive value. VTE-BLEED score: Venous Thrombo-Embolism Bleed; RIETE: Registro informatizado de la enfermedad tromboembólica en España; Computerized Registry of Patients with Venous Thromboembolism; ORBIT: Outcomes Registry for Better Informed Treatment; HAEMORR_2_HAGES score: Hepatic or Renal Disease, Ethanol Abuse, Malignancy, Older Age, Reduced Platelet Count or Function, Re-Bleeding, Hypertension, Anemia, Genetic Factors, Excessive Fall Risk and Stroke; ATRIA score: Anticoagulation and Risk Factors in Atrial Fibrillation; HAS-BLED score: Hypertension, Abnormal Renal/Liver Function, Stroke, Bleeding History or Predisposition, Labile International Normalized Ratio, Elderly, Drugs/Alcohol.

**Figure 3:**
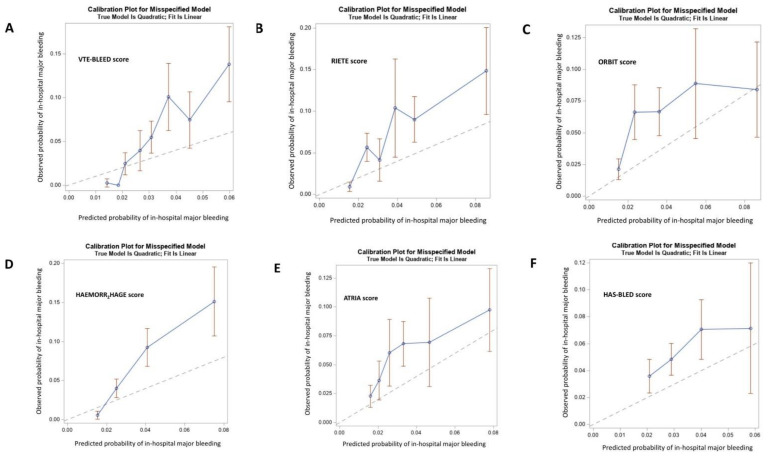
Decile calibration plots of six in-hospital major bleeding prediction risk scores. All six scores were not well calibrated with the predicted risks and their confidence intervals were not distributed around the observed in-hospital bleeding risks. (**A**) VTE-BLEED score; (**B**) RIETE score; (**C**) ORBIT score; (**D**) HAEMORR_2_HAGES score; (**E**) ATRIA score; (**F**) HAS-BLED score.

**Figure 4:**
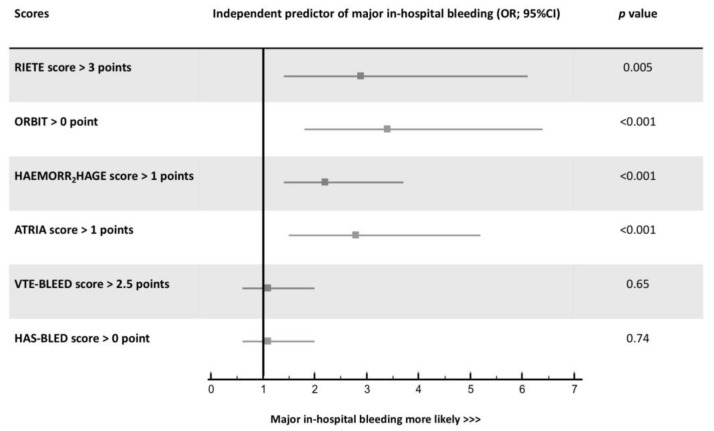
Adjusted-threshold 2-level category major bleeding scores as predictors of in-hospital major bleeding after multivariable adjustment. OR: odds ratio; CI: confidence interval.

Overall, the RIETE score had the best global model fit with the lowest BIC and AIC and the highest Nagelkerke’s R^2^. Harrell’s c index ranged from 0.570 with the HAS-BLED to 0.692 with the RIETE score (Table 4). Figure 2 displays the ROC curves of the six early major bleeding scores as well as their corresponding sensitivity, specificity, PPV, and NPV based on the 2-level risk categories. The RIETE score had a numerically higher Harrell’s c-index than the HAEMORR_2_HAGES, ORBIT, ATRIA, and VTE-BLEED bleeding scores. Reclassification parameters (i.e., IDI and NRI) were significantly higher with the RIETE, ORBIT, and ATRIA scores than the others. The HAS-BLED score had the lowest discriminatory and reclassification capacities (Table 5). All bleeding scores were not well calibrated with the predicted risks and their confidence intervals were not distributed around the observed early bleeding risks (Figure 3). ROC assessment for dichotomy isolation of the bleeding risk scores are displayed in Supplementary Figure S1. The 2-level RIETE, ORBIT, ATRIA, and HAEMORR_2_HAGES scores were independently associated with early major bleeding after multi-variable adjustment (Figure 4).

### 3.3. Sensitivity Analysis

In total, 2621 patients (95.2%) had non-high risk PE and 120 patients (4.3%) were treated with advanced therapy (3.8% with systemic thrombolysis, and 0.5% with surgical embolectomy). Discrimination performances of all bleeding scores were similar across patients with non-high risk PE and those who did not receive advanced therapy (Supplementary Figure S2).

## 4. Discussion

In our multicenter cohort analysis, the RIETE score had better global fit and higher discriminatory and reclassification capacities compared to the other available bleeding-prediction scores for the assessment of early major bleeding risk after an acute PE. However, the accuracy of the RIETE score remains low with corresponding sensitivity and specificity of 65.8% and 66.4%, respectively, generating high rates of both false positives and false negatives in the bleeding risk appraisal. Since bleeding risk assessment is of importance in the acute phase of PE with a well-demonstrated close relationship between bleeding events and early mortality [5,6,7], the development of a dedicated early risk score is crucial.

The RIETE score was validated in 15,206 patients from the RIETE registry treated with three months of anticoagulation for the treatment of PE with a fair c-index of 0.719 (95% CI, 0.689–0.749) [29]. To the best of our knowledge, the present cohort study is the largest evaluating performances of available bleeding risk scores for the prediction of in-hospital bleeding. Our results are consistent with and strengthen those reported by Klok et al., who showed low risk prediction accuracy of the Kuijer, the RIETE, the HAEMORR_2_HAGES, the HAS-BLED, and the ATRIA scores with c-indices ranging from 0.57 to 0.64 (c-index for the RIETE score, 0.60 (95 % CI, 0.47–0.72)) in 655 patients from the single-center PERGO registry [16]. Our results regarding prediction performance of the VTE-BLEED score are the opposite of those recently reported in 655 patients. In this single center cohort, the authors observed a higher c-index (i.e., 0.69 (95% CI, 0.58–0.80)) than ours for the discrimination capacity of this score to predict in-hospital major bleeding, as well as an independent relationship between VTE-BLEED score and in-hospital bleeding events after multivariable adjustment. Differences in study design (i.e., exclusion of patients receiving reperfusion therapy in the aforementioned study) and a nearly 4-fold lower sample size may explain these discrepancies [6].

We observed almost similar discrimination capacity of the ORBIT score as compared to the RIETE score in our analysis (Harrell’s c-index, 0.681 vs 0.692), with a high sensitivity of the ORBIT score when collapsed into 2-level categories (84.1%). Nevertheless, the associated low specificity of 43.8% renders the ORBIT score useless in clinical practice with a related high rate of false positives and a corresponding low rate of true negatives for low-risk bleeding risk prediction. Nevertheless, bleeding risk scores in the acute phase of PE should probably not be derived from AF populations as patient characteristics differ widely from PE patient characteristics. For instance, mean age was 69.8 years in 17,162 AF patients versus 60.2 years in 11,842 VTE patients in large international registries [30,31].

The accurate identification of acute PE patients at high risk of bleeding with the use of bleeding prediction scores, together with individualized decision-making may prompt alternative therapeutic strategies. Low molecular weight heparin may be a preferred option rather than unfractionated heparin in high bleeding risk patients, to avoid supratherapeutic anticoagulation when advanced therapy is planned [32]. Direct oral anticoagulants have been shown to be associated with a lower risk of bleeding than the standard heparin and vitamin K antagonist regimen [33]. The bleeding risk of patients treated with systemic thrombolysis can potentially be overcome by the use of alternative reperfusion strategies, such as ultrasound-facilitated catheter-based therapy or surgical embolectomy [34,35,36,37]. The 2019 ESC guidelines recommend inferior vena cava filter implantation for patients with an absolute contraindication to anticoagulant therapy, based on a lower risk of recurrent PE over the first month compared with patients not receiving this device [3,38]. Finally, the identification of high bleeding risk patients should prompt providers to mitigate other modifiable risk factors such as concomitant anti-platelet therapy or hypertension [39]. The recent development of a dedicated in-hospital risk score may help to fill this gap [40].

The strengths of the present study include the prospective patient recording in different centers, the high rate of consecutive inclusions (99.9%), the independent adjudication of clinical end-points, and the robustness of statistical approaches. In contrast, therapeutic decision making was left to the discretion of the treating physicians. Thus, the type of initial anticoagulation and measures for anticoagulation quality control were not standardized. We similarly applied the ISTH criteria for the definition of major bleeding events and did not evaluate other bleeding definitions such as the GUSTO or CRUSADE definitions [41,42]. Finally, although the rate of events (specifically major bleeding) in the overall population was low, which may be a limitation, it is nonetheless similar to rates reported in other publications [6,16]. 

## 5. Conclusions

Among six available bleeding risk scores, the RIETE score had the best performance profile. However, the accuracy of the RIETE score remains low, generating high rates of both false positives and false negatives in the bleeding risk appraisal. 

## Figures and Tables

**Table 1:** Baseline characteristics and in-hospital management of 2754 study patients according to the occurrence or not of early major bleeding.

	All Study Patients (*n* = 2754)	Missing Values (%)	Patients without Major Bleeding (*n* = 2672)	Patients with Major Bleeding (*n* = 82)	*p* Value
Age, year	67.3 ± 17.4	0	67.2 ± 17.4	70.2 ± 14.4	0.12
Female sex (%)	1414 (51.3)	0	1362 (50.1)	52 (63.4)	0.03
BMI, kg/m^2^	27.4 ± 5.9	0.2	27.4 ± 5.9	27.8 ± 7.4	0.59
Comorbidities (%)					
Hypertension	1300 (47.2)	0.1	1258 (47.1)	42 (51.2)	0.45
Diabetes mellitus	366 (13.3)	0.2	354 (13.2)	12 (14.6)	0.71
Chronic pulmonary disease	236 (8.6)	0.3	23 (8.6)	5 (6.1)	0.41
Coronary artery disease	378 (13.7)	0.2	365 (13.7)	13 (15.5)	0.56
Active cancer ^a^	507 (18.4)	0.1	490 (18.3)	17 (20.7)	0.58
Prior stroke	166 (6.0)	0.1	157 (5.9)	9 (11.0)	0.09
Prior VTE	654 (23.8)	0	631 (23.6)	23 (3.5)	0.35
Prior bleeding	42 (1.5)	0.3	38 (1.4)	4 (4.9)	0.07
Recent surgery ^b^	192 (7.0)	0.2	181 (6.8)	11 (13.4)	0.02
Concomitant medication usage predisposing to bleeding ^c^	112 (4.1)	0.1	102 (3.8)	10 (12.2)	0.008
Antiplatelet therapy	93 (3.4)	-	17 (0.6)	3 (3.7)	-
Anticoagulant	20 (0.7)	-	86 (3.2)	7 (8.5)	-
Low-risk for long-term recurrence	702 (25.6)	0.3	678 (25.4)	24 (29.3)	0.42
Associated DVT	1120 (40.7)	1.1	1082 (40.5)	38 (46.3)	0.30
Clinical characteristics					
HR at admission, bpm	89.9 ± 19.1	0.6	89.8 ± 19.1	94.1 ± 9.8	0.04
SBP at admission, mmHg	137.7 ± 23.6	0.1	138.0 ± 23.4	131.6 ± 24.0	0.01
SaO_2_ at admission, %	93.4 ± 5.6	1.1	93.5 ± 5.3	90.4 ± 9.9	<0.001
Biological data					
Hemoglobin (g/dL)	13.3 ± 2.9	1.0	13.4 ± 2.8	11.1 ± 2.8	<0.001
eGRF_CKD-EPI_, mmol/L	75.9 ± 24.9	0.9	76.3 ± 24.9	62.2 ± 24.9	<0.001
Positive troponin	981 (35.6)	0.9	938 (35.1)	43 (52.4)	0.002
Echo data					
RV dysfunction	911 (33.1)	1.1	871 (32.6)	40 (48.8)	0.003
sPESI (median, Q1–Q3)	2 (1–3)	0.9	2 (1–3)	3 (2–3)	0.009
ESC-defined risk PE category (%)					<0.001
Low-risk	443 (16.1)	-	438 (16.4)	5 (6.1)	
Intermediate-low risk	1594 (57.9)	-	1550 (58.0)	44 (53.7)	
Intermediate-high risk	584 (21.2)	-	563 (21.1)	21 (25.6)	
High-risk	133 (4.8)	-	121 (4.5)	12 (14.6)	
Bleeding scores (median, Q1–Q3)					
VTE-BLEED score	2.5 (1.5–3.5)	-	2.5 1 (1.5–3.5)	3.0 (2.5–4.5)	<0.001
RIETE score	3 (2–4)	-	1 (0–2)	3.5 (3–4.5)	<0.001
ORBIT score	1 (0–2)	-	1 (0–2)	2 (1–3)	<0.001
HAEMORR_2_HAGES score	1 (0–1)	-	1 (0–2)	2 (1–2)	<0.001
ATRIA score	1 (0–3)	-	1 (0–3)	3 (2–5)	
HAS-BLED score	1 (0–1)	-	1 (0–1)	1 (1–2)	0.01
In-hospital treatments (%)					
Anticoagulation					
UFH	603 (21.9)	-	559 (20.9)	44 (53.7)	<0.001
LMWH/fondaparinux	1538 (42.3)	-	1501(56.2)	39 (44.0)	<0.001
DOAC	613 (22.2)	-	612 (22.9)	1 (1.2)	<0.001
Reperfusion therapy					
Thrombolysis	107 (3.9)	-	98 (3.7)	9 (11.0)	<0.001
Surgical embolectomy	13 (0.5)	-	7 (0.3)	6 (7.3)	<0.001
ECMO	17 (0.6)	-	9 (0.3)	8 (9.8)	<0.001
Inferior vena cava filter	9 (0.3)	-	7 (0.3)	2 (2.4)	<0.001

BMI: body mass index; VTE: venous thromboembolism; DVT: deep vein thrombosis; HR: heart rate; b.*p*.m: beat per minute; SBP: systolic blood pressure; Sa: oxygen saturation; eGRF_CKD-EPI_: estimated glomerular function by using the Chronic Kidney Disease Epidemiology Collaboration equation; RV: right ventricle; sPESI: simplified Pulmonary Embolism Severity Index; UFH: unfractionated heparin; LMWH: low molecular weight heparin; DOAC: direct oral anticoagulant; ECMO: extra-corporeal membrane oxygenation. ^a^ Active or anti-tumor therapy within the last 6 months, or metastatic state according to the 2019 European Society of Cardiology guidelines. ^b^ Within the past 4 weeks. ^c^ Antiplatelet therapy, non-steroidal anti-inflammatory drug. VTE-BLEED score: Venous Thrombo-Embolism Bleed; RIETE: Registro informatizado de la enfermedad tromboembólica en España; Computerized Registry of Patients with Venous Thromboembolism; ORBIT: Outcomes Registry for Better Informed Treatment; HAEMORR_2_HAGES score: Hepatic or Renal Disease, Ethanol Abuse, Malignancy, Older Age, Reduced Platelet Count or Function, Re-Bleeding, Hypertension, Anemia, Genetic Factors, Excessive Fall Risk and Stroke; ATRIA score: Anticoagulation and Risk Factors in Atrial Fibrillation; HAS-BLED score: Hypertension, Abnormal Renal/Liver Function, Stroke, Bleeding History or Predisposition, Labile International Normalized Ratio, Elderly, Drugs/Alcohol; ESC-defined risk PE category: pulmonary embolism risk category according to the guidelines of the European Society of Cardiology.

**Table 2:** Univariable and multivariate predictors of early major bleeding, length of stay and in-hospital all-cause mortality.

Variable	OR (95% CI)	*p* Value	OR (95% CI)	*p* Value
	Univariate Analysis		Multivariate Analysis	
Major Bleeding				
Female sex	1.5 (1.04–2.1)	0.03	-	-
Age > 80 (years)	1.6 (1.04–2.5)	0.03	-	-
Weight < 60 (kg)	1.6 (1.01–2.6)	0.04		
Recent surgery ^a^	2.1 (1.1–4.1)	0.02	-	-
Concomitant medication usage predisposing to bleeding ^b^	2.8 (1.4–5.4)	0.004	2.3 (1.1–4.7)	0.02
Syncope	3.5 (2.0–6.1)	<0.001	2.4 (1.3–4.3)	0.004
Heart rate > 80 (b.p.m)	1.9 (1.1–3.2)	0.02	1.8 (1.1–3.1)	0.03
Arterial oxyhemoglobin saturation < 90 (%)	1.5 (1.0–2.4)	0.05	-	-
Positive troponin ^c^	2.0 (1.3–3.2)	0.001	-	-
Platelet count < 150,000/mm^3^)	2.0 (1.3–3.3)	0.007	-	-
GFR _CKD-EPI_^d^ < 60 (mL/min)	2.0 (1.2–3.3)	0.007	1.8 (1.1–2.9)	0.03
Hemoglobin < 12 (g/dL)	4.6 (2.9–7.2)	<0.001	3.7 (2.3–6.1)	<0.001
RV dysfunction ^e^	1.9 (1.3–3.1)	0.002	-	-
Systemic thrombolysis during in-hospital phase	3.2 (1.6–6.7)	0.001	-	-
Length of stay				
Age > 80 years	2.3 (1.9–2.6)	<0.001	1.9 (1.6–2.2)	<0.001
Female sex	1.3 (1.1–1.5)	<0.001		
Hypertension	1.4 (1.2–1.6)	<0.001		
Diabetes mellitus	1.4 (1.2–1.7)	<0.001		
Chronic pulmonary disease	2.0 (1.6–2.5)	<0.001	2.0 (1.6–2.6)	<0.001
Coronary artery disease	1.4 (1.2–1.7)	<0.001		
Prior stroke	2.0 (1.6–2.7)	<0.001		
Cognitive disorders	1.6 (1.1–2.3)	0.009		
Concomitant medication usage predisposing to bleeding ^b^	1.6 (1.1–2.3)	0.008		
Associated DVT	1.4 (1.3–1.6)	<0.001	1.4 (1.2–1.6)	<0.001
Syncope	1.7 (1.3–2.1)	<0.001		
PAS < 90 mmHg at admission	1.6 (1.2–2.2)	0.002		
Heart rate > 110 b.p.m	1.7 (1.4–1.9)	<0.001	1.6 (1.3–1.9)	<0.001
RV dysfunction at admission ^d^	1.6 (1.4–1.9)	<0.001		
Positive troponin ^b^	2.4 (2.1–2.7)	<0.001	2.0 (1.7–2.3)	<0.001
GFR _CKD-EPI_ < 60 mL/min ^c^	1.9 (1.7–2.2)	<0.001		
Hemoglobin at admission < 12 g/dL	1.4 (1.2–1.6)	<0.001		
In-hospital major bleeding	4.8 (3.2–7.1)	<0.001	4.2 (2.9–5.8)	<0.001
In-hospital death				
Age > 80 years	1.8 (1.2–2.7)	0.002	-	-
BMI (per kg^−1^)	1.1 (1.02–1.2)	0.003	-	-
Coronary artery disease	2.3 (1.5–3.6)	<0.001	2.1 (1.2–3.7)	0.01
Active cancer	4.7 (3.2–6.8)	<0.001	6.9 (4.0–11.9)	<0.001
Prior VTE	0.5 (0.3–0.8)	0.009	-	-
Associated DVT	0.6 (0.3–0.8)	0.002	-	-
Syncope	2.6 (1.5–4.2)	<0.001	-	-
PAS < 90 mmHg at admission	6.7 (4.1–10.7)	<0.001	4.0 (1.8–3.7)	<0.001
Heart rate > 110 b.p.m	1.9 (1.2–2.9)	0.003	-	-
RV dysfunction at admission ^d^	1.7 (1.2–2.4)	0.005	-	-
Positive troponin ^b^	2.7 (1.9–4.0)	<0.001	2.9 (1.7–4.9)	<0.001
GFR _CKD-EPI_ < 60 mL/min ^c^	3.2 (2.2–4.7)	<0.001	-	-
Hemoglobin at admission < 12 g/dL	3.9 (2.7–5.7)	<0.001	-	-
In-hospital major bleeding	9.1 (5.3–15.6)	<0.001	8.4 (4.0–17.6)	<0.001

CI: confidence interval; OR: odds ratio; BMI: body mass index; RV: right ventricle. ^a^ Within the past 4 weeks. ^b^ Antiplatelet therapy, non-steroidal anti-inflammatory drug. ^c^ Glomerular filtration rate calculated with the Chronic Kidney Disease Epidemiology Collaboration (CKD-EPI) formula. ^d^ Defined as a value > 99th percentile of healthy subjects with a coefficient of variation of 10%. ^e^ Defined by the presence of at least one of the following on echography: increased end-diastolic right ventricle/left ventricle diameter > 1.0 in the apical four-chamber view, flattened intraventricular septum, decrease tricuspid annular plane systolic excursion < 16 mm, or right heart thrombus detected in right heart cavities.

**Table 3:** Frequency of observed early major bleeding according to the risk categories of bleeding prediction scores.

	RIETE Score		ORBIT Score		HAEMORR_2_HAGES Score		ATRIA Score		VTE-BLEED Score		HAS-BLED Score	
3-level categories	Number of major bleeding/number of patients (%, 95% CI)											
Low risk	-	-	54/2371	2.3%; (1.7–3.0)	35/1943	1.8%; (1.2–2.5)	56/2314	2.4%; (1.8–3.1)	-	-	17/837	2.0%; (1.2–3.2)
Intermediate risk	50/2150	2.3%; (1.71–3.0)	13/169	7.7%; (4.1–12.8)	44/764	5.8%; (4.2–7.7)	4/100	4.0%; (1.1–9.3)	-	-	57/1805	3.2%; (2.4–4.1)
High risk	32/604	5.3%; (3.6–7.4)	15/199	7.0%; (4.0–11.3)	3/47	6.4%; (1.3–17.6)	22/318	6.5%; (4.1–9.7)	-	-	8/112	7.1%; (3.1–13.4)
2-level risk categories *	Number of major bleeding/number of patients (%, 95% CI)											
Low risk	28/1803	1.5%; (1.0–2.2)	13/1183	1.1%; (0.6–1.9)	35/1943	1.8%; (1.3–2.5)	20/1389	1.5%; (0.8–2.1)	24/1405	1.7%; (1.1–2.5)	17/837	2.0%; (1.2–3.2)
High risk	54/951	5.7%; (4.3–7.4)	69/1571	4.4%; (3.4–5.5)	47/811	5.8%; (4.3–7.6)	62/1365	4.5%; (3.5–7.4)	58/1349	4.3%; (3.2–5.5)	65/1917	3.4%; (2.2–4.3)

* Optimized cut-off values for 2-level categories. Analysis: RIETE score >3 point; ORBIT > 0 point; HAEMORR_2_HAGES score > 1 points; ATRIA score > 1 points; VTE-BLEED score > 2.5 points; HAS-BLED score > 0 point.

**Table 4:** Global model fit and discrimination of bleeding scores for the prediction of early major bleeding.

Score	VTE-BLEED	RIETE	ORBIT	HAEMORR_2_HAGES	ATRIA	HAS-BLED
Overall model fit						
BIC	817.4	721.2	722.1	728.2	726.4	746.7
AIC	734.5	709.3	710.3	716.4	714.6	734.8
Nagelkerke’s R^2^ (%)	4.81	4.89	4.85	3.91	4.19	1.08
Discrimination						
Harrell’s c index	0.633	0.692	0.681	0.674	0.669	0.570

AIC: Akaike information criterion; BIC: Bayes information criterion.

**Table 5:** C-statistics, integrated discrimination improvement, and net reclassification improvement of bleeding scores for in-hospital major bleeding discrimination and reclassification.

Score Comparison	∆C-Index (%)	*p* Value	IDI (%) (95% CI)	*p* Value	NRI (%) (95% CI)	*p* Value
HAEMORR_2_HAGES vs. RIETE	0.2 (−5.2–5.6)	0.94	−0.1 (−0.5–0.3)	0.57	1.4 (−19.2–22.1)	0.90
ORBIT vs. RIETE	1.7 (−0.1–0.3)	0.73	1.2 (−0.1–0.3)	0.36	−3.7 (−15.2–7.8)	0.74
ATRIA vs. RIETE	2.3 (−3.3–5.6)	0.62	0.2 (−0.9–0.5)	0.18	7.1 (−8.9–23.1)	0.63
VTE-BLEED vs. RIETE	3.3 (−1.3–7.9)	0.15	0.4 (0.2–0.7)	<0.001	58.1 (44.2–72.2)	<0.001
HAS-BLED vs. RIETE	9.6 (5.1–134.0)	<0.001	0.8 (0.6–1.2)	<0.001	58.2 (44.2–72.2)	<0.001
HAEMORR_2_HAGES vs. ORBIT	−0.3 (−0.5–0.5)	0.88	−0.2 (−0.6–0.1)	0.28	−2.6 (−22.6–17.4)	0.81
ATRIA vs. ORBIT	1.6 (−2.5–3.6)	0.72	0.8 (−0.1–0.3)	0.37	2.1 (−10.0–14.4)	0.19
VTE-BLEED vs. ORBIT	2.7 (−2.0–7.6)	0.26	0.3 (0.06–0.6)	0.001	55.8 (39.6–72.1)	<0.001
HAS-BLED vs. ORBIT	8.9 (3.8–14.1)	<0.001	0.8 (0.5–1.0)	<0.001	55.8 (39.6–72.1)	<0.001
ATRIA vs. HAEMORR_2_HAGES	0.9 (−4.8–6.6)	0.74	0.3 (−0.1–0.7)	0.14	−6.5 (−19.5–18.2)	0.95
VTE-BLEED vs. HAEMORR_2_HAGES	3.1 (−2.0–8.3)	0.22	0.5 (0.2–0.9)	0.002	9.8 (–7.2–26.8)	0.87
HAS-BLED vs. HAEMORR_2_HAGES	9.3 (3.6–15.1)	0.001	1.0 (0.6–1.4)	<0.001	57.4 (35.7–79.1)	<0.001
VTE-BLEED vs. ATRIA	2.2 (−3.7–8.1)	0.46	0.2 (−0.09–0.5)	0.14	53.7 (34.7–72.7)	<0.001
HAS-BLED vs. ATRIA	2.9 (2.6–14.3)	0.004	0.7 (0.4–1.0)	<0.001	53.7 (34.7–72.6)	<0.001
HAS-BLED vs. VTE-BLEED	6.2 (0.4–12.0)	0.03	0.4 (0.2–0.7)	<0.001	44.8 (24.7–64.9)	<0.001

Δ: delta (change); IDI: integrated discrimination improvement; NRI: net reclassification improvement.

## Data Availability

All the data used and/or analyzed in this study are reported in the results and supplementary material and are available from the corresponding author on reasonable written request.

## Supplementary Materials

The following are available online at https://www.mdpi.com/article/10.3390/jcm10163615/s1, Figure S1: Receiving operative curve assessment for dichotomy isolation of the bleeding risk scores; Figure S2: Sensitivity analysis with receiving operative curves, and the corresponding C-indices of bleeding risk scores (i.e., ORBIT, HAEMORR2AGE, ATRIA, HAS-BLED, VTE-BLEED, and RIETE scores) in non-high-risk PE patients (*n* = 2621) (A) and in patients who did not received advanced therapy (*n* = 2634) (B); Table S1: ORBIT, HAEMORR_2_HAGES, ATRIA, HAS-BLED, VTE-BLEED, and RIETE scores, and staging systems for risk of major bleeding complications.
