# Supplementary material for: Available Bleeding Scoring Systems Poorly Predict Major Bleeding in the Acute Phase of Pulmonary Embolism
Source: J Clin Med. 2021 Aug 16;10(16):3615. doi: 10.3390/jcm10163615 (PMC8396851; doi:10.3390/jcm10163615)
Supplement: Supplementary file 1 [file jcm-10-03615-s001.zip › jcm-1300733-supplementary.pdf]

Supplementary material

Table S1. ORBIT, HAEMORR^{2}HAGES, ATRIA, HAS-BLED, VTE-BLEED, and RIETE scores, and staging systems for risk of major bleeding complications [1-5].

**ORBIT score**	**Points**
Age > 74 years	+1
Bleeding history (i.e. Any history of GI bleeding, intracranial bleeding, or hemorrhagic stroke)	+2
GFR <60 mL/min/1.73 m^2	+1
Treatment with antiplatelet agents	+1
**HEMORR^{2}HAGES score**	**points**
Hepatic or Renal Disease	+1
Ethanol (Alcohol) Abuse	**+1**
Age > 75 years	+1
Reduced Platelet Count or Function Includes aspirin use, any thrombocytopenia or blood dyscrasia, like hemophilia	+1
History of past bleeding	+2
Hypertension (uncontrolled)	+1
Anemia Hb <13 g/dL for Men; Hb <12 g/dL for Women	+1
Genetic Factors CYP 2C9 single-nucleotide polymorphisms	+1
Excessive fall risk	+1
Stroke history	+1
**ATRIA score**	**Points**
Anemia Hb <13 g/dL (Male), <12 g/dL (Female)	+3
Severe Renal Disease Glomerular filtration rate <30 mL/min or dialysis-dependent	+3
Age > 74 years	+2
Any Prior Hemorrhage Diagnosis (e.g. GI bleed, intracranial hemorrhage)	+1
Hypertension history	+1
**HAS-BLED score**	**Points**
Hypertension Uncontrolled, >160 mmHg systolic	+1
Renal disease Dialysis, transplant, Cr >2.26 mg/dL or >200 µmol/L	+1
Liver disease Cirrhosis or bilirubin >2x normal with AST/ALT >3x normal	+1
Stroke history	+1
Prior major bleeding or predisposition to bleeding	+1

Labile INR<br>Unstable/high INRs, time in therapeutic range <60%	+1
Age > 65 years	+1
Medication usage predisposing to bleeding<br>Aspirin, clopidogrel, NSAIDs	+1
Alcohol use<br>≥8 drinks/week	+1
**VTE-BLEED score**	**Points**
Active cancer	+ 2
Male Patient with Uncontrolled Hypertension<br>[Systolic BP ≥140mm Hg]	+1
Anemia<br>[Hb <130g/L Men. Hb <120g/L Women]	+ 1.5
History of Bleeding<br>[Major or non-major clinically relevant bleeding]	+ 1.5
Renal Dysfunction<br>[CrCl 30-60 ml/min]	+ 1.5
Age ≥60 years	+ 1.5
**RIETE score**	**Points**
Recent Major Bleeding	+ 2
Malignancy	+ 1
Anaemia<br>[Hb <130g/L Men. Hb <120g/L Women]	+ 1.5
Renal Dysfunction<br>[Creatinine <1.2 mg/dL]	+ 1.5
Clinically overt PE	+1
Age >75 yrs	+ 1

ORBIT score: Points are assigned for the presence of each variable. The sum of the variable points produces the total point score (ORBIT score; range, 0-7). ORBIT risk staging increased with point totals: low-risk (< 2 points), intermediate-risk (3 point), and high-risk (4-7 points). HEMORR^{2}HAGES score: Points are assigned for the presence of each variable. The sum of the variable points produces the total point score (HEMORR^{2}HAGES score; range, 0-12). ORBIT risk staging increased with point totals: low-risk (< 2 points), intermediate-risk (3 point), and high-risk (4-7 points). Atria score: Points are assigned for the presence of each variable. The sum of the variable points produces the total point score (Atria score; range, 0-10). Atria risk staging increased with point totals: low-risk (< 4 points), intermediate-risk (4 point), and high-risk (> 4 points). HAS-BLED score: Points are assigned for the presence of each variable. The sum of the variable points produces the total point score (HAS-BLED score; range, 0-9). HAS-BLED risk staging increased with point totals: relatively low-risk (0-1 points), intermediate-risk (3 point), and high-risk (> 3 points). VTEBLEED score: Points are assigned for the presence of each variable. The sum of the variable points produces the total point score (VTE-BLEED score; range, 0-9). VTE-BLEED risk staging increased with point totals: low-risk (0-1points), intermediate-risk (2, 3 points) or high-risk (≥ 3 points). RIETE score: Points are assigned for the presence of each variable. The sum of the variable points produces the total point score (RIETE risk score; range, 0–8). RIETE risk staging increased with point totals: low-risk (0 points), intermediate-risk (1-4 points), or high-risk (>4 points). Abbreviations: GI, gastro-intestinal; Hb, hemoglobin; CR, creatinine; AST, Aspartate aminotransférase ; ALT, Alanine aminotransférase ; INR, international

normalized ratio; NSAIDs, Non-steroidal anti-inflammatory drugs ; CrCl, creatinine clearance; PE, pulmonary embolism.

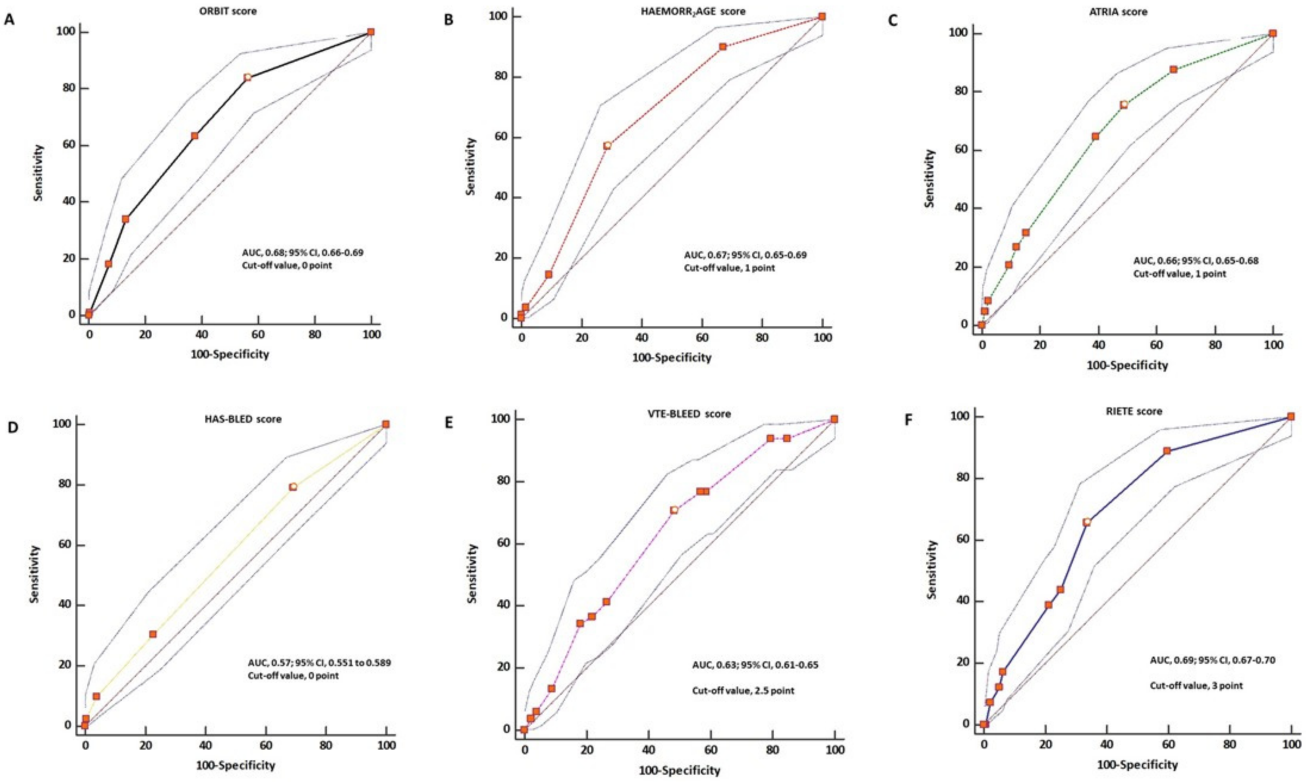

Figure S1: Receiving operative curve assessment for dichotomy isolation of the bleeding risk scores (A, ORBIT score; B, HAEMORR$_2$AGE score; C, ATRIA score; D, HAS-BLED score; E, VTE-BLEED score, and F, RIETE score). ORBIT score: Points are assigned for the presence of each variable. The sum of the variable points produces the total point score (ORBIT score; range, 0-7). ORBIT risk staging increased with point totals: low-risk (< 2 points), intermediate-risk (3 point), and high-risk (4-7 points). HEMORR^{2}HAGES score: Points are assigned for the presence of each variable. The sum of the variable points produces the total point score (HEMORR^{2}HAGES score; range, 0-12). ORBIT risk staging increased with point totals: low-risk (< 2 points), intermediate-risk (3 point), and high-risk (4-7 points). Atria score: Points are assigned for the presence of each variable. The sum of the variable points produces the total point score (Atria score; range, 0-10). Atria risk staging increased with point totals: low-risk (< 4 points), intermediate-risk (4 point), and high-risk (> 4 points). HAS-BLED score: Points are assigned for the presence of each variable. The sum of the variable points produces the total point score (HAS-BLED score; range, 0-9). HAS-BLED risk staging increased with point totals: relatively low-risk (0-1 points), intermediate-risk (3 point), and high-risk (> 3 points). VTEBLEED score: Points are assigned for the presence of each variable. The sum of the variable points produces the total point score (VTE-BLEED score; range, 0-9). VTE-BLEED risk staging increased with point totals: low-risk (0-1points), intermediate-risk (2, 3 points) or high-risk (≥ 3 points). RIETE score: Points are assigned for the presence of each variable. The sum of the variable points produces the total point score (RIETE risk score; range, 0–8). RIETE risk staging increased with point totals: low-risk (0 points), intermediate-risk (1-4 points), or high-risk (>4 points).

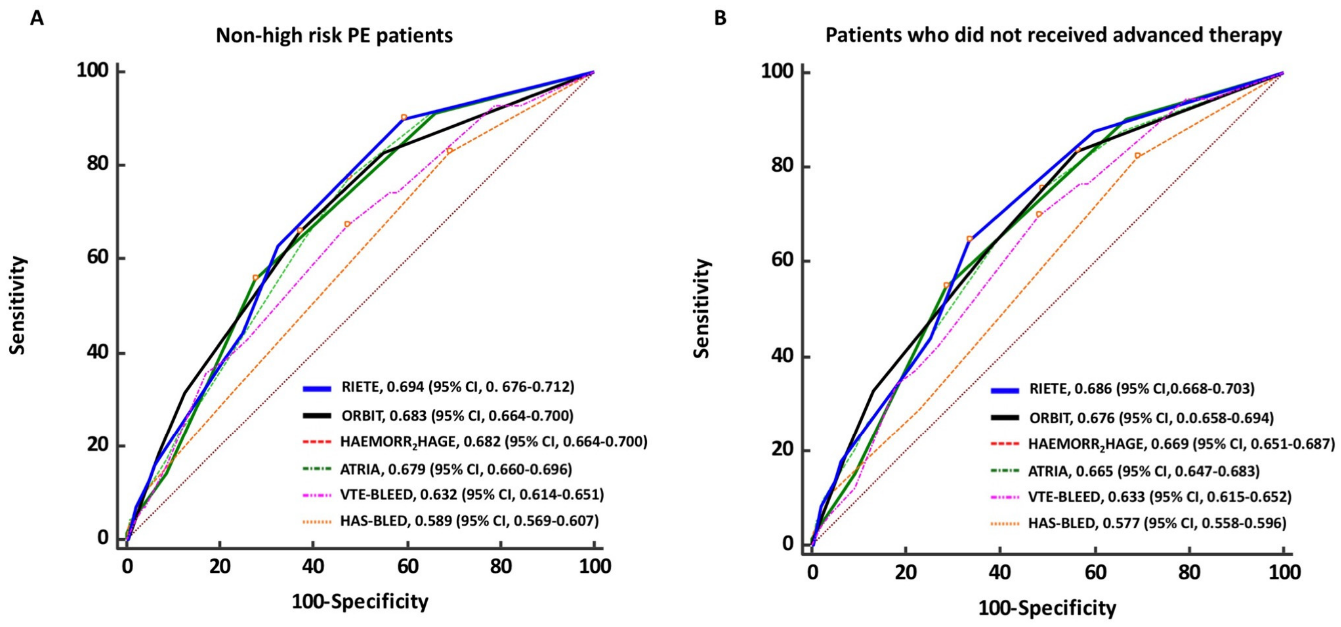

Figure S2. Sensitivity analysis with receiving operative curves, and the corresponding C-indices of bleeding risk scores (i.e. ORBIT, HAEMORR$_2$AGE, ATRIA, HAS-BLED, VTE-BLEED, and RIETE scores) in non-high-risk PE patients (*n* = 2,621) (A) and in patient who did not received advanced therapy (*n*=2,634) (B). PE: pulmonary embolism.

References

1. Ruiz-Gimenez, N.; Suarez, C.; Gonzalez, R.; Nieto, J.A.; Todoli, J.A.; Samperiz, A.L.; Monreal, M.; Investigators, R. Predictive variables for major bleeding events in patients presenting with documented acute venous thromboembolism. Findings from the RIETE Registry. *Thromb Haemost* **2008**, *100*, 26–31, doi:10.1160/TH08-03-0193.
2. Klok, F.A.; Hosel, V.; Clemens, A.; Yollo, W.D.; Tilke, C.; Schulman, S.; Lankeit, M.; Konstantinides, S.V. Prediction of bleeding events in patients with venous thromboembolism on stable anticoagulation treatment. *Eur Respir J* **2016**, *48*, 1369-1376, doi:10.1183/13993003.00280–2016.
3. O'Brien, E.C.; Simon, D.N.; Thomas, L.E.; Hylek, E.M.; Gersh, B.J.; Ansell, J.E.; Kowey, P.R.; Mahaffey, K.W.; Chang, P.; Fonarow, G.C.; et al. The ORBIT bleeding score: a simple bedside score to assess bleeding risk in atrial fibrillation. *Eur Heart J* **2015**, *36*, 3258–3264, doi:10.1093/eurheartj/ehv476.
4. Gage, B.F.; Yan, Y.; Milligan, P.E.; Waterman, A.D.; Culverhouse, R.; Rich, M.W.; Radford, M.J. Clinical classification schemes for predicting hemorrhage: results from the National Registry of Atrial Fibrillation (NRAF). *Am Heart J* **2006**, *151*, 713–719, doi:10.1016/j.ahj.2005.04.017.
5. Fang, M.C.; Go, A.S.; Chang, Y.; Borowsky, L.H.; Pomernacki, N.K.; Udaltsova, N.; Singer, D.E. A new risk scheme to predict warfarin-associated hemorrhage: The ATRIA (Anticoagulation and Risk Factors in Atrial Fibrillation) Study. *J Am Coll Cardiol* **2011**, *58*, 395–401, doi:10.1016/j.jacc.2011.03.031.
